# Trajectories and Influencing Factors of Online Health Information–Seeking Behaviors Among Community-Dwelling Older Adults: Longitudinal Mixed Methods Study

**DOI:** 10.2196/77549

**Published:** 2025-11-05

**Authors:** Ting Wang, Qian Dong, Qun Sun, Hui Dong, Xiaolong Bu, Yanan Wang, Yanni Su, Cuiping Liu

**Affiliations:** 1 School of Nursing, Shandong First Medical University & Shandong Academy of Medical Sciences, No.619, Changcheng Road, Taian, Shandong Province, 271000, China, 86 1-385-421-8960; 2 Shandong Provincial Chronic Disease Hospital, Qingdao, Shandong Province, China; 3 Nursing Department, Shandong Provincial Taishan Hospital, Taian, Shandong Province, China

**Keywords:** aged, digital health, information-seeking behaviors, internet, quantitative research, qualitative research

## Abstract

**Background:**

The internet has emerged as a critical avenue for the public to access health information resources. As online health information is a key resource for older adults, the factors influencing their online health information–seeking behaviors (OHISBs) are crucial.

**Objective:**

This study aims to examine the trajectories and influencing factors of OHISB among community-dwelling older adults and to explore their attitudes toward this information in daily contexts.

**Methods:**

A longitudinal mixed methods study was conducted involving 346 older adults from three communities in Shandong Province, China. In the quantitative phase, data were collected at three time points: baseline (T1), 6 months postbaseline (T2), and 12 months postbaseline (T3). Latent class growth modeling and logistic regression identified trajectories and predictors of OHISB, while a cross-lagged panel model analyzed longitudinal relationships among digital health literacy (DHL), technology anxiety (TA), and OHISB. Subsequently, we conducted semistructured, in-depth interviews with 16 older adults from different OHISB trajectory subgroups using a descriptive phenomenological approach. Quantitative and qualitative results were integrated via triangulation.

**Results:**

Latent class growth modeling identified three OHISB trajectories: low-level declining group (92/346, 26.6%), medium-level stable group (187/346, 54%), and high-level declining group (67/346, 19.4%). Multivariate logistic regression identified household registration, education, income, chronic disease status, internet frequency and duration, online health information attitude and experience, DHL, and TA as significant predictors of OHISB trajectory. TA mediated the relationship between DHL and OHISB, with an effect size of 0.04 (SE 0.014, 95% CI 0.0123–0.068). Qualitative interviews with 16 participants revealed four themes: personal cognition, emotional experience, external environment, and behavioral choices. The quantitative study elucidated the pathway between DHL, TA, and OHISB, providing a data-driven foundation for the qualitative inquiry. In a complementary manner, the qualitative study revealed themes not captured quantitatively—particularly how personal factors influence behavior through attitudes—and elaborated on sources of social support and the role of the medical environment. Together, both methods convergently demonstrate that older adults’ information behavior is not static but shaped by individual and environmental factors, resulting in distinct behavioral patterns.

**Conclusions:**

Both quantitative and qualitative findings clarified the developmental process of OHISB among older adults in communities and the important effects of DHL, TA, and risk perception on OHISB. Although self-efficacy, health anxiety, self-perceived aging, social support, and health care environment were not addressed in the quantitative study, they emerged as important factors shaping older adults’ OHISB in qualitative interviews. Personalized intervention measures should target various OHISB trajectory characteristics and their influencing factors to enhance the health conditions of community-dwelling older adults.

## Introduction

Rapid aging of populations and the digital transformation of health care systems present intertwined challenges and opportunities for public health. In China, adults aged 60 years and above account for 22% of the population, with 15.6% aged 65 years or older [1], underscoring the urgency to address age-related health needs [2]. Concurrently, the Digital China Initiative aims to integrate advanced technologies into public services, including health care [3], positioning digital health information as a primary resource. While digital platforms offer significant potential for managing health [4,5], older adults face multiple barriers to their use, including difficulties with digital tools, psychological reluctance, and physiological limitations like vision impairment [6]. Despite their heightened health demands due to physiological decline and chronic disease burdens [7], older adults exhibit low motivation, infrequent engagement, and distrust toward online health information. This mismatch between digital health information potential and real-world utilization highlights the importance of understanding the dynamics of older adults’ online health information–seeking behaviors (OHISBs).

Previous studies have identified distinct trajectories in health-related behaviors among older adults, such as sleep patterns [8] and frailty [9]. Individuals likely differ in OHISB due to varying characteristics and environments [10]. A scoping review of information-seeking behaviors among information consumers [11] categorizes the trajectory of information-seeking behaviors into three patterns: remaining stable, increasing, and decreasing. Yet, analogous research focusing on older adults is notably absent. Evidence from younger groups (eg, university students) suggests notable behavioral differences [12], leading us to hypothesize that older adults may also exhibit diverse OHISB trajectories. Understanding these trajectories is essential for developing precise interventions customized to the specific requirements of subgroups within the aging population.

Various factors influence OHISB in older adults, including personal traits (eg, physical and cognitive decline, limited education, inadequate digital health literacy [DHL]) [13], and psychological components like technology anxiety (TA), marked by a negative emotional reaction to technology [14]. Environmental factors include inadequate social support [15] and an online landscape characterized by misinformation, information overload, and *information cocoons* [16]. DHL, the capacity to seek, evaluate, and apply digital health information [17], is positively associated with OHISB [18]. Conversely, TA negatively impacts the perceived ease of using the internet and the willingness to use it [19]. While existing studies have established the reciprocal influence among DHL, TA, and OHISB [18,20], the specific connections among these factors are still unclear.

The study was conducted based on three theoretical frameworks. First, Longo’s Extended Model of Health Information–Seeking Behavior [21] posits that OHISB is shaped by personal attributes and environmental contexts. Second, the Cognitive-Affective-Conative Theory [22] elucidates how cognitive factors influence affective responses, which in turn drive behavioral intentions. Finally, Bandura’s Social Cognitive Theory [23] emphasizes triadic reciprocity among personal, behavioral, and environmental factors, providing a holistic lens to analyze OHISB dynamics.

Furthermore, given that OHISB is a complex and multidimensional concept, studies on OHISB believe that qualitative research has made it possible to learn more about users’ search behaviors and to comprehend participants’ experiences, internal motivations, thoughts, and feelings [24]. Therefore, this study employed a longitudinal mixed methods design. The specific objectives of this study were as follows: (1) to identify the heterogeneous trajectories of OHISB among community-dwelling older adults and analyze the influencing factors across different trajectories; (2) to explore the pathway relationships among DHL, TA, and OHISB in older adults; and (3) to conduct semistructured interviews to investigate the multidimensional factors affecting older adults’ OHISB and their patterns of information-seeking behaviors. By integrating quantitative and qualitative data, this study not only advances the understanding of older adults’ OHISB but also provides actionable insights for developing targeted interventions, such as tailored digital literacy training, age-friendly information design, and enhanced social support systems, to promote digital health equity among older populations.

## Methods

### Study Design

This study employed a sequential explanatory mixed methods design. Quantitative data were collected at three time points and analyzed first, followed by qualitative data collection and analysis. The qualitative phase identified heterogeneous OHISB trajectory groups, addressing the “what” of behavioral patterns. Next, based on the Cognitive–Affective–Conative framework [22], a mediation effect model is constructed to examine the mediating role of TA between DHL and OHISB, revealing the psychological mechanisms (ie, the “why”) behind different trajectories. Guided by both the Extended Model of Health Information–Seeking Behavior and Social Cognitive Theory [21,23], findings were integrated via triangulation [25].

### Ethical Considerations

Ethical approval was obtained from the Medical Ethics Committee of Shandong First Medical University (R202310130170). Prior to participation, all participants were fully informed about the study’s purpose, procedures, potential risks, and benefits. Written informed consent was obtained from each participant. The research process consistently adhered to the principles of informed consent, voluntariness, confidentiality, and nonmaleficence, with all data deidentified and used solely for academic purposes. Participants retained the right to withdraw from the study at any time. Each participant was given a Chinese ¥20 (¥1=US $0.14) gift upon the completion of each assessment as a gesture of appreciation and to cultivate a positive rapport with them.

### Quantitative Data

#### Participants

The longitudinal survey took place between September 2023 and January 2025, with participants recruited through convenience sampling method from two communities in Tai’an City, Shandong Province, and one community in Linyi City. Criteria for inclusion were as follows: age ≥60 years, residency in the community for ≥6 months, possessing adequate communication skills, and providing informed consent. Exclusion criteria comprised severe physical or acute illnesses, complete loss of activities of daily living, psychiatric disorder diagnosis among participants, and lack of prior experience using digital devices. The first survey wave (T1) commenced in September 2023, encompassing 346 older adults residing in the community, with 243 participants from the Tai’an community and 103 participants from the Linyi community. By the 6-month follow-up (T2), 327 participants had responded, and at the 12-month follow-up (T3), the number decreased to 303, resulting in a 12.43% attrition rate, which was lower than the 20% threshold. Data collection details are shown in Multimedia Appendix 1.

#### Data Collection and Procedures

Based on Longo’s expanded model of health information–seeking behaviors, this study primarily explores the impact of personal factors on OHISB among community-dwelling older adults. Data on personal factors mainly consist of four key components: demographic factors (such as gender, age, household registration, marital status, dwelling state, number of children, employment status, education level, income level, and medical insurance), health-related conditions (including chronic disease status, self-assessment of health status, and degree of health concern), internet usage (internet usage frequency and internet usage duration), and the utilization of online health information (attitudes toward online health information, willingness to seek online health information, and experience in seeking online health information). The DHL scale [26] assessed the DHL level of the older adults. TA was assessed using the TA scale, translated and validated in Chinese by Sun et al [27]. OHISB was assessed using a modified version of the OHISB scale developed by Wang et al [28], adapted to older adults’ current behavioral patterns and influencing factors. All scales demonstrated good reliability and validity.

After establishing contact with community directors, the research objectives and content were explained to them. With their assistance, we utilized the community resident information management system to identify potential participants. This system allowed us to screen for older adults who met the study’s eligibility criteria based on demographic and health profiles. Information gathered from three community directors revealed that around 600 participants met the study criteria. Subsequently, these potential participants were informed in person or via telephone about the study and their preliminary eligibility. Surveys were conducted daily, with community staff notifying 20‐30 eligible participants who met the criteria each time. The participation rate averaged at about 65%. At the baseline, the participants who met the inclusion and exclusion criteria completed the questionnaires face-to-face under the guidance of two well-trained researchers. Questionnaires included the Personal Information Questionnaire, DHL scale, TA scale, and OHISB scale. The questionnaires were collected immediately upon completion and reviewed on-site to identify any missing or inaccurate responses. Following the baseline questionnaire survey, small gifts were given to participants, and contact details were retained to foster trust and rapport. Subsequent surveys at T2 and T3 involved completing the scales at T1, excluding the Personal Information Questionnaire.

### Statistical Analysis

All questionnaire data were analyzed using IBM SPSS Statistics (Version 27.0; IBM Corp) and Mplus (Version 8.3; Muthen & Muthen). A 2-tailed significance level of *α*=.05 was applied, with *P*<.05 considered statistically significant. The Harman single-factor test addressed potential common method bias [29]. Pearson correlations examined longitudinal associations among DHL, TA, and OHISB. Latent class growth modeling (LCGM) was used to identify heterogeneous trajectories of OHISBs among community-dwelling older adults. The optimal model was selected based on theoretical plausibility, interpretability, and fit indices, including the Akaike information criterion, Bayesian information criteria (BIC), and sample-size adjusted BIC, where lower values indicate better fit; entropy (range 0‐1), with values ≥0.8 suggesting over 90% classification accuracy; and the Bootstrap Likelihood Ratio Test and Lo–Mendell–Rubin Likelihood Ratio Test, which were employed to compare model fit differences across varying class solutions, where *P*<.05 indicates that the *k*-class model significantly outperforms the (*k*-1)-class model [30]. Subgroups with sample sizes <5% were deemed nonrepresentative and excluded [31]. For missing data, Full Information Maximum Likelihood estimation was applied to iteratively estimate parameters using available information [32]. *χ*² tests and one-way ANOVA were used to compare demographic, DHL, and TA variables across latent subgroups. Variables with *P*<.05 were included in multivariate logistic regression to identify the factors that influence the developmental trajectory of heterogeneity in OHISB. A cross-lagged panel model was employed to explore the longitudinal relationship between DHL, TA, and OHISB, and the model was estimated using Maximum Likelihood Estimate. Model fit was assessed using the chi-square-to-degree-of-freedom ratio (*χ*²/*df*), comparative fit index (>0.9), Tucker–Lewis index (>0.9), root mean square error of approximation (<0.08), and standardized root mean square residual (<0.08) [33]. If model fit was unsatisfactory, modification indices guided adjustments. Bootstrapping with 2000 resamples was performed to assess the longitudinal mediating role of TA between DHL and OHISB, with 95% CIs excluding 0 indicating significance.

### Qualitative Data

#### Participants

For the interview, a stratified sampling method was employed to select community-dwelling older adults from different trajectory subgroups who had participated fully in the longitudinal study. The recruitment process was as follows: during the initial survey, participants were asked if they would be willing to be contacted for a potential follow-up interview. Subsequently, from this pool of willing participants, we purposefully selected individuals based on the principle of maximum variation sampling to ensure representation across key demographic and behavioral characteristics. The sample size was determined based on thematic saturation, with recruitment continuing until no new content or themes emerged from the integrated interview data. The final sample consisted of 16 community-dwelling older adults for formal interviews.

#### Data Collection and Procedures

The co–first authors (TW and QD) conducted qualitative data collection through face-to-face semistructured interviews after the longitudinal survey in January 2025. The interviews focused on factors influencing OHISB among older adult community members. They adhered to a preestablished topic guide (available in Multimedia Appendix 2) designed by the research team, and 2 participants were selected for the preliminary interviews. Sessions took place in a quiet, well-lit meeting room at the community health center, with each interview lasting approximately 40 minutes. To ensure interview quality, the interviewer facilitated in-depth responses by actively listening, timely questioning, and adjusting question sequencing. Additionally, interviewees were allowed to make audio recordings and take notes.

#### Data Analysis

Following each interview, audio recordings were transcribed verbatim within 24 hours and imported into NVivo 15 (Lumivero) for analysis. For the analysis, two team members (QD and CL), who have experience in qualitative research, employed Colaizzi’s seven-step phenomenological analysis method [34]. The process included (1) immersive data reading, (2) extraction of significant statements, (3) coding and categorization, (4) theme identification, (5) contextualization within lived experiences, (6) synthesis of essential phenomenological structures, and (7) returning analyzed findings to participants for validation to ensure credibility. This iterative approach ensured rigor in capturing the nuanced dynamics of OHISB among older adults.

### Integration of Mixed Methods Data

We integrated the quantitative and qualitative findings using triangulation, a classic method for data integration in mixed methods research. Specifically, we formulated a triangulation protocol, listing each significant discovery of both quantitative and qualitative findings, and developed a joint display table that juxtaposed the key findings from both datasets according to the theoretical domains (eg, individual, environmental, behavioral). We then systematically examined this table to identify points of convergence (where findings from both methods confirmed each other), complementarity (where findings from one method elaborated or clarified findings from the other), and discordance (where findings diverged). This structured comparison allowed us to discern overarching patterns and develop a more comprehensive understanding than either approach could yield alone.

## Results

### Quantitative Analysis

#### Descriptive Statistics of Participants and Correlation Analysis of Main Variables

Descriptive statistics of participants are presented in Multimedia Appendix 3. Nearly half of the participants (169/346, 48.8%) were aged 66‐75 years, with a mean age of 68.37 (SD 5.58) years. Over half were female (185/346, 53.5%) and had urban household registration (203/346, 58.7%). Educational level varied, with smaller proportions reporting primary education (67/346, 19.4%) or college-level education and above (51/346, 14.7%). A majority had chronic diseases (188/346, 54.3%), while fewer reported a high degree of health concern (131/346, 37.9%). Regarding internet use frequency, most participants used the internet occasionally (126/346, 36.4%) or sometimes (137/346, 39.6%). Although 59.8% (207/346) expressed willingness to seek online health information, only 40.8% (141/346) had experience in seeking online health information. Comparative analysis revealed no significant differences between completers and dropouts in demographics or key variables (*χ*^2^/*t*=−0.06 to 4.99, *P*=.08-.96). Scores and correlations for DHL, TA, and OHISB across time points are shown in Table 1. Mean scores for DHL at T1–T3 were 36.23 (SD 8.47), 35.94 (SD 8.07), and 35.10 (SD 8.79), respectively; TA scores were 37.48 (SD 5.63), 37.02 (SD 6.64), and 36.47 (SD 7.28), respectively; and OHISB scores were 55.76 (SD 11.80), 55.86 (SD 11.84), and 54.32 (SD 12.89), respectively. DHL was positively correlated with OHISB (0.540<*r*<0.743, *P*<.01) and negatively correlated with TA (−0.530<*r*<−0.281, *P*<.01). TA also negatively correlated with OHISB (−0.485<*r*<−0.273, *P*<.01).

**Table 1.** Descriptive analysis and correlational analysis (*r*) of T1-T3 digital health literacy, technology anxiety, and online health information–seeking behaviors.

	Mean (SD)	DHL^a^ T1	DHL T2	DHL T3	TA^b^ T1	TA T2	TA T3	OHISB^c^ T1	OHISB T2	OHISB T3
DHL T1	36.23 (8.47)	—^d^	0.796^e^	0.647^e^	−0.303^e^	−0.322^e^	−0.309^e^	0.685^e^	0.650^e^	0.620^e^
DHL T2	35.94 (8.07)	0.796^e^	—	0.749^e^	−0.281^e^	−0.460^e^	−0.396^e^	0.617^e^	0.656^e^	0.649^e^
DHL T3	35.10 (8.79)	0.647^e^	0.749^e^	—	−0.281^e^	−0.417^e^	−0.530^e^	0.540^e^	0.583^e^	0.743^e^
TA T1	37.48 (5.63)	−0.303^e^	−0.281^e^	−0.281^e^	—	0.493^e^	0.351^e^	−0.290^e^	−0.332^e^	−0.378^e^
TA T2	37.02 (6.64)	−0.322^e^	−0.460^e^	−0.417^e^	0.493^e^	—	0.664^e^	−0.273^e^	−0.385^e^	−0.472^e^
TA T3	36.47 (7.28)	−0.309^e^	−0.396^e^	−0.530^e^	0.351^e^	0.664^e^	—	−0.275^e^	−0.343^e^	−0.485^e^
OHISB T1	55.76 (11.80)	0.685^e^	0.617^e^	0.540^e^	−0.290^e^	−0.273^e^	−0.275^e^	—	0.840^e^	0.723^e^
OHISB T2	55.86 (11.84)	0.650^e^	0.656^e^	0.583^e^	−0.332^e^	−0.385^e^	−0.343^e^	0.840^e^	—	0.806^e^
OHISB T3	54.32 (12.89)	0.620^e^	0.649^e^	0.743^e^	−0.378^e^	−0.472^e^	−0.485^e^	0.723^e^	0.806^e^	—

a DHL: digital health literacy.

b TA: technology anxiety.

c OHISB: online health information–seeking behaviors.

d Not available.

e *P*<.01.

#### Developmental Trajectories of OHISB

**Figure 1.**
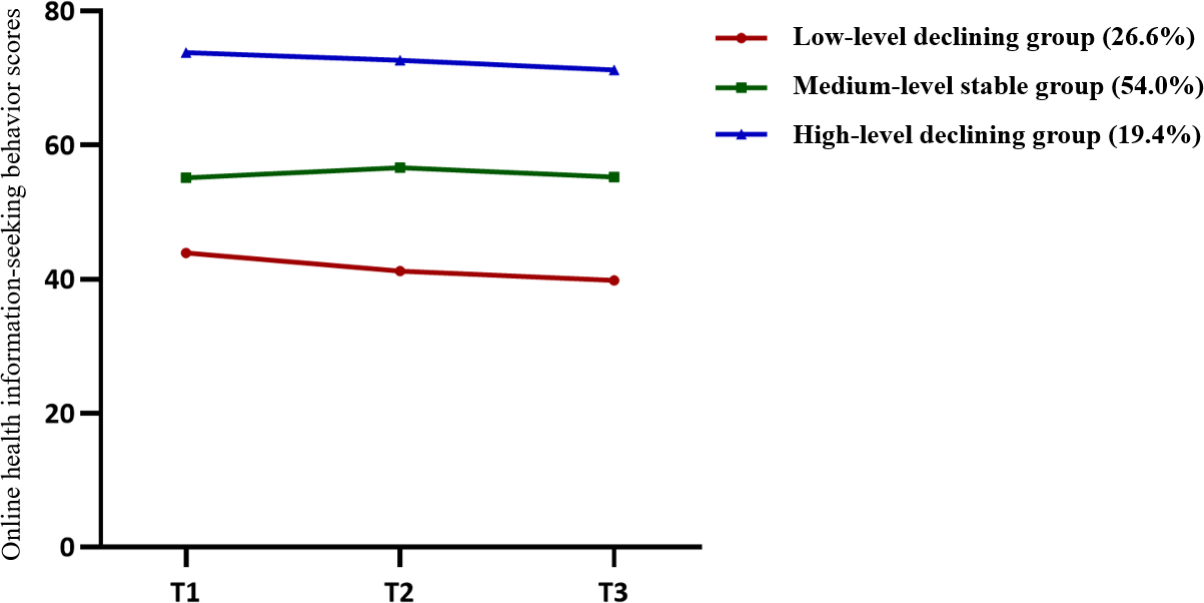
The heterogeneous developmental trajectories of online health information–seeking behaviors among older adults.

LCGM was applied to fit 1‐5 trajectory classes. As shown in Multimedia Appendix 4, the 3-class model demonstrated the best fit, with the lowest Akaike Information Criterion, BIC, and sample-size adjusted BIC values, highest entropy (>0.8, indicating >90% classification accuracy), and significant Lo–Mendell–Rubin Likelihood Ratio Test and Bootstrap Likelihood Ratio Test results (*P*<.001). All subgroups exceeded the 5% sample size threshold. The three trajectories were characterized as follows (Figure 1 and Multimedia Appendix 5): low-level declining group (92/346, 26.6%) showed initial low OHISB scores followed by a decline; medium-level stable group (187/346, 54%) exhibited initial moderate OHISB scores with stability over time; and high-level declining group (67/346, 19.4%) displayed initial high OHISB scores followed by a decline.

#### Influencing Factors of Heterogeneous OHISB Trajectories

Group differences across trajectories are summarized in Multimedia Appendix 6. Significant differences were observed in age, household registration, employment status, education level, income level, chronic disease status, degree of health concern, internet usage frequency, internet usage duration, attitude toward online health information, willingness to seek online health information, experience in seeking online health information, DHL, and TA (*χ*^2^/*F*=7.44‐139.28, *P*=.001-.02). Multivariate logistic regression (Table 2) identified household registration, education level, income level, chronic disease status, internet usage frequency, internet usage duration, attitude toward online health information, experience in seeking online health information, DHL, and TA as significant influencing factors of OHISB trajectory (*β*=−2.64 to 1.72, *P*=.001-.048). The assignment method of each type of variable is shown in Multimedia Appendix 7. Compared to the low-level declining group, the medium-level stable group had higher odds of elevated DHL (odds ratio [OR] 1.215, 95% CI 1.150‐1.348), while the former was associated with primary education (OR 0.089, 95% CI 0.015‐0.537), no chronic diseases (OR 0.344, 95% CI 0.151‐0.784), internet use <5 years (OR 0.368, 95% CI 0.138‐0.986), and unsure attitudes toward online health information (OR 0.226, 95% CI 0.080‐0.640). Compared to the low-level declining group, the high-level declining group had higher odds of urban household registration (OR 5.555, 95% CI 1.516‐20.353) and elevated DHL (OR 1.522, 95% CI 1.350‐1.715), while the former was linked to primary education (OR 0.029, 95% CI 0.002‐0.354), income <¥2000 per month (OR 0.064, 95% CI 0.005‐0.861), absence of chronic diseases (OR 0.260, 95% CI 0.079‐0.852), occasional internet use (OR 0.015, 95% CI 0.001‐0.344), no online health-seeking experience (OR 0.175, 95% CI 0.043‐0.719), and higher TA (OR 0.885, 95% CI 0.795‐0.986). Compared to the medium-level stable group, the high-level declining group showed higher odds of urban residence (OR 3.329, 95% CI 1.185‐9.350) and elevated DHL (OR 1.222, 95% CI 1.117‐1.337), while the former was linked to middle school education (OR 0.271, 95% CI 0.074‐0.987), income ¥2000‐¥4000 per month (OR 0.314, 95% CI 0.123‐0.801), no online health-seeking experience (OR 0.199, 95% CI 0.075‐0.528), and higher TA (OR 0.766, 95% CI 0.832‐0.968). Lower internet use frequency (seldom: OR 0.071, 95% CI 0.006‐0.887; occasionally: OR 0.140, 95% CI 0.035‐0.558; sometimes: OR 0.229, 95% CI 0.069‐0.759) was more common in the medium-level stable group.

**Table 2.** Multiple logistic regression analysis of the influential factors of diverse trajectories of online health information–seeking behaviors among older adults in communities.

Variables	C2^a^ VS C1^b^^,^^c^			C3^d^ VS C1^c^			C3 VS C2^e^		
	B	*P* value	Odds ratio (95% CI)	B	*P* value	Odds ratio (95% CI)	B	*P* value	Odds ratio (95%CI)
Age									
≤65 y	1.134	.08	3.107 (0.867-11.138)	0.867	.33	2.380 (0.423-13.394)	−0.267	.67	0.766 (0.222-2.640)
66-75 y	1.016	.10	2.761 (0.821-9.290)	0.960	.26	2.611 (0.497-13.722)	-0.056	.93	0.946 (0.284-3.143)
>75 y (Ref^f^)	—^g^	—	—	—	—	—	—	—	—
Household registration									
Urban	0.512	.22	1.669 (0.731-3.811)	1.715	.01	5.555 (1.516-20.353)	1.203	.02	3.329 (1.185-9.350)
Rural (Ref)	—	—	—	—	—	—	—	—	—
Employment status									
Unemployed/job seeking	−0.900	.18	0.407 (0.108-1.531)	−1.093	.46	0.335 (0.018-6.134)	−0.193	.89	0.824 (0.059-11.550)
Employed/employed before retirement	—	—	—	—	—	—	—	—	—
Education level									
Primary school	−2.416	.01	0.089 (0.015-0.537)	−3.556	.01	0.029 (0.002-0.354)	−1.140	.23	0.320 (0.050-2.030)
Middle school	−0.673	.43	0.510 (0.096-2.699)	−1.980	.06	0.138 (0.017-1.098)	−1.307	.05	0.271 (0.074-0.987)
High school/vocational school/polytechnic school	−0.448	.65	0.639 (0.091-4.476)	−0.689	.54	0.502 (0.056-4.522)	−0.241	.67	0.786 (0.259-2.380)
College degree or above (Ref)	—	—	—	—	—	—	—	—	—
Income level									
<¥2000/mo^h^	−1.027	.12	0.358 (0.097-1.328)	−2.748	.04	0.064 (0.005-0.861)	−1.721	.14	0.179 (0.018-1.765)
¥2000-¥4000/mo	−0.119	.84	0.888 (0.285-2.768)	−1.276	.08	0.279 (0.066-1.186)	−1.157	.02	0.314 (0.123-0.801)
>¥4000/mo (Ref)	—	—	—	—	—	—	—	—-	—
Chronic disease status									
No	−1.068	.01	0.344 (0.151-0.784)	−1.347	.03	0.260 (0.079-0.852)	−0.279	.53	0.757 (0.314-1.820)
Yes (Ref)	—	—-	—	—	—	—	—	—	—
Degree of health concern									
Concerned	0.145	.78	1.157 (0.411-3.253)	0.916	.23	2.499 (0.562-11.107)	0.770	.18	2.161 (0.708-6.591)
Moderate	0.209	.68	1.232 (0.464-3.276)	−0.011	.99	0.989 (0.205-4.773)	−0.220	.73	0.803 (0.227-2.834)
No concern (Ref)	—	—	—	—	—	—	—	—	—
Internet usage frequency									
Seldom	−1.557	.12	0.211 (0.029-1.526)	−4.201	.01	0.015 (0.001-0.344)	−2.644	.04	0.071 (0.006-0.887)
Occasionally	0.056	.95	1.057 (0.181-6.162)	−1.907	.09	0.148 (0.017-1.326)	−1.963	.01	0.140 (0.035-0.558)
Sometimes	0.576	.54	1.778 (0.288-10.968)	−0.897	.41	0.408 (0.049-3.431)	−1.472	.02	0.229 (0.069-0.759)
Often (Ref)	—	—	—	—	—	—	—	—	—
Internet usage duration									
<5 y	−0.999	.05	0.368 (0.138-0.986)	−0.559	.45	0.572 (0.136-2.403)	0.439	.43	1.552 (0.523-4.606)
≥5 y (Ref)	—	—	—	—	—	—	—	—	—
Attitude toward online health information									
Trust	0.448	.45	1.566 (0.484-5.061)	0.730	.41	2.076 (0.367-11.755)	0.282	.67	1.326 (0.361-4.872)
Unsure	−1.488	.01	0.226 (0.080-0.640)	−0.050	.96	0.952 (0.150-6.035)	1.438	.07	4.213 (0.887-20.003)
Distrust (Ref)	—	—	—	—	—	—	—	—	—
Willingness to seek for online health information									
No	−0.247	.56	0.781 (0.341-1.791)	−0.291	.67	0.747 (0.199-2.803)	−0.045	.93	0.956 (0.332-2.756)
Yes (Ref)	—	—	—	—	—	—	—	—	—
Experience in seeking online health information									
No	−0.126	.82	0.882 (0.304-2.562)	−1.742	.02	0.175 (0.043-0.719)	−1.617	.001	0.199 (0.075-0.528)
Yes (Ref)	—	—	—	—	—	—	—	—	—
Digital health literacy	0.219	<.001	1.245 (1.150-1.348)	0.420	<.001	1.522 (1.350-1.715)	0.201	<.001	1.222 (1.117-1.337)
Technology anxiety	−0.014	.73	0.986 (0.911-1.067)	−0.122	.03	0.885 (0.795-0.986)	−0.108	.005	0.766 (0.832-0.968)

a C2: medium-level stable group.

b C1: low-level declining group.

c Low-level declining group as reference.

d C3: high-level declining group.

e Medium-level stable group as reference.

f Ref: reference.

g Not available.

h ¥1=US $0.14.

#### Longitudinal Mediating Role of TA Between DHL and OHISB

**Figure 2.**
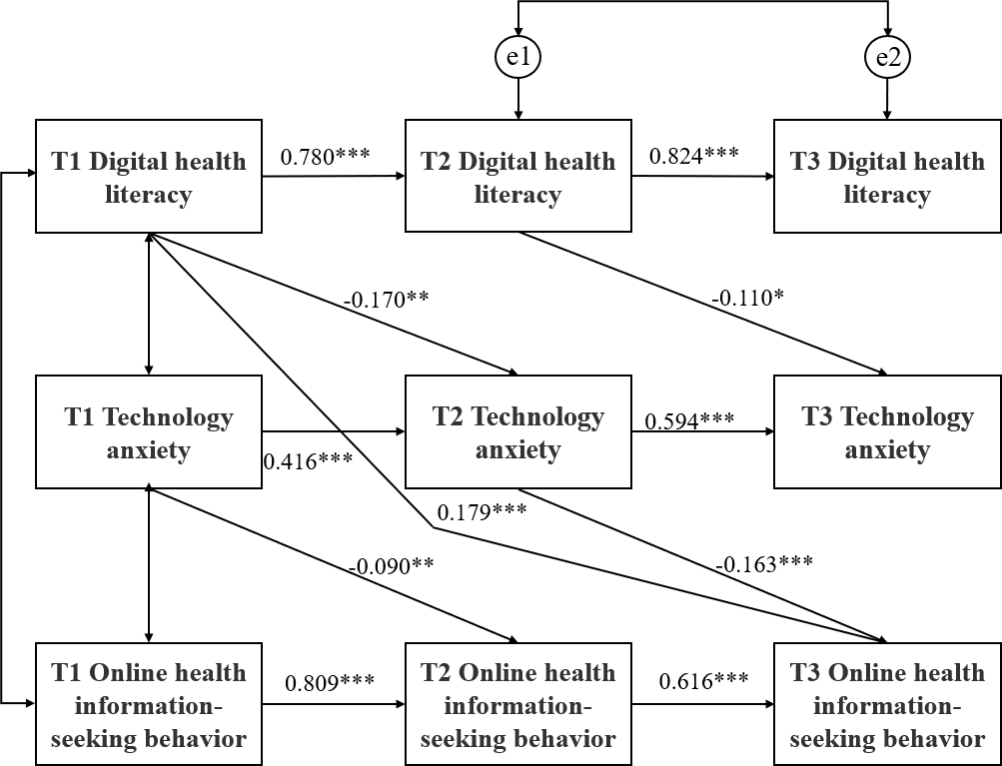
Results of the mediation analysis in cross-lagged panel model. ***P<.001, **P<.01, *P<.05.

A cross-lagged panel mediation model was constructed with DHL as the independent variable, TA as the mediator, and OHISB as the dependent variable. The initial model was poorly fitted to the observed data. Based on the Modified Index prompt, which allows for error correlation of the same observed variables at three time points [97], the path of error correlation for DHL at time points T2 and T3 was added. The revised model achieved good fit: *χ*²/*df*=2.90, comparative fit index=0.985, Tucker–Lewis index=0.966, root mean square error of approximation=0.074, and standardized root mean square residual=0.063. Path analysis revealed (Figure 2) that T1 DHL negatively predicted T2 TA (*β*=−.170, *P*<.01); T2 TA negatively predicted T3 OHISB (*β*=−.163, *P*<.001); and T1 DHL positively predicted T3 OHISB (*β*=.179, *P*<.001). Bootstrap analysis (2000 resamples) confirmed a significant direct effect of T1 DHL on T3 OHISB (effect=0.259, SE 0.059, 95% CI 0.143‐0.374) and a significant indirect effect mediated by T2 TA (effect=0.040, SE 0.014, 95% CI 0.012‐0.068), supporting the longitudinal mediation hypothesis.

### Qualitative Analysis

#### Overview

A total of 16 community-dwelling older adults were interviewed, including 7 men and 9 women, with an average age of 67.06 (SD 4.28) years. Each subgroup was numbered as D, Z, and G, respectively, based on their developmental trajectories of OHISB. Detailed information about the respondents can be found in Multimedia Appendix 8. Through the analysis and synthesis of the interview data from 16 participants, this study identified 4 central themes, 10 higher order themes, and 23 lower order themes, as detailed in Table 3.

**Table 3.** Overview of themes and categories.

Social cognitive theory, Central themes, Higher order themes	Lower order themes		
	Low-level declining group	Medium-level stable group	High-level declining group
Personal factors			
Personal cognition			
Health literacy	Inadequate health literacy	Lack of digital health literacy	Adequate health and digital health literacy
Self-efficacy	Low self-efficacy	Low self-efficacy	High self-efficacy
Risk perception	Low risk perception	High risk perception	High risk perception
Emotional experience			
Health anxiety	Low health anxiety	High health anxiety	High health anxiety
Technology anxiety	Technology nervousness and fear	Technology fear	Privacy and security concerns
Self-perceived aging	Strong aging perception	Moderate aging perception	Positive aging perception
Environment factors			
External environment			
Social support	Inadequate social support	Inadequate social support	Good social support
Health care environment	—^a^	—	Complicated offline medical procedures; poor medical service quality
Behavior modes			
Behavioral choices			
Information avoidance	Cognitive avoidance	Cognitive avoidance; risk avoidance	Risk avoidance
Information seeking	Proxy seeking	Proactive seeking; proxy seeking	Proactive seeking

a Not available.

#### Personal Cognition

The personal cognition was attributed to three aspects: health literacy, self-efficacy, and risk perception. Specifically, older adults in the low-level declining group reported inadequate health literacy:

I understand my physical condition. I eat well and sleep well, so I simply don’t need to know all that health information.

[No.D2]

Those in the medium-level stable group reported a lack of DHL:

I’m old and don’t really follow health-related public accounts on my phone. My daughter tried to teach me before, but I forgot how to use them soon after.

[No.Z3]

The older adults in the high-level declining group reported adequate health literacy and DHL:

My spouse has hypertension. I check information about high blood pressure on WeChat moments and public accounts to better monitor their condition.

[No.G1]

Low self-efficacy was reported by older adults in the low-level declining and medium-level stable groups:

The online health information is excessively technical, making it difficult for me to comprehend.

[No.D1]

Seniors in the high-level declining group reported high self-efficacy:

I disseminate beneficial health information in group chats for my children’s perusal. If the content is novel even to them, it elicits a sense of pride within me.

[No.G3]

Older adults in the low-level declining group reported low risk perception:

If I get sick, I’ll go to the hospital—small issues to small clinics, serious ones to big hospitals. I’m old anyway; how many more years can I live? What’s the use of knowing so much health info?

[No.D4]

Older adults in the medium-level stable group and high-level declining group reported high risk perception:

Having undergone heart surgery last year, it is crucial for me to prioritize my health to prevent a recurrence of such a procedure. Online health resources have helped me learn so much I never knew before.

[No.Z2]

#### Emotional Experience

Emotional experience was linked to three factors: health anxiety, TA, and self-perceived aging. Notably, older adults in the low-level declining group exhibited low health anxiety:

I don’t have any major health issues. No need to learn more about health information.

[No.D3]

The older adults in the medium-level stable group and the high-level declining group reported high levels of health anxiety:

I didn’t understand things like myocardial infarction before, but several old coworkers have gotten it recently. I heard smoking increases the risk, so I looked up prevention methods online.

[No.Z4]

The older adults in the low-level declining group reported technology nervousness:

My son wants to buy me a wristband to monitor blood pressure and heart rate. But will I even know how to use it

[No.D1]

Older adults in the low-level declining and medium-level stable groups reported fear of technology

We seniors only use basic WeChat. Checking health information online is too complicated—we can’t learn how.

[No.D4]

Those in the high-level declining group reported privacy and security concerns:

Searching for health info online is good, but sometimes I click the wrong thing and end up on other pages. Not sure if it’s safe.

[No.G2]

The older adults in the low-level declining group reported strong aging perception:

Aging naturally brings health problems. Knowing more health info won’t change that.

[No.D5]

The older adults in the medium-level stable group reported moderate aging perception:

With hypertension and heart disease, I have to be extra careful at my age. Online health info helps manage my conditions.

[No.Z4]

The high-level declining group of older adults was informed of positive aging perception:

Learning health info online isn’t harmful. It says seniors should exercise more, so I do tai chi every morning. I’m even healthier now than before retirement—maybe fitter than some young folks!

[No.G1]

#### External Environment

The external environment was characterized by social support and the health care environment. Older adults in the low-level declining and medium-level stable categories reported inadequate social support:

My son is so busy with work even after coming home. How can I bother him with questions?

[No.D3]

Those in the high-level declining group reported good social support:

If I don’t understand online health information, I ask my grandchildren to explain it.

[No.G3]

Seniors in the high-level declining group reported complicated offline medical procedures:

Hospital visits are exhausting—queues for registration, medicine, scans…Now I check things online first. When I must go, I can even book appointments online

[No.G3]

and poor experience of medical service quality:

Managing numerous patients leaves little room for effective communication with a doctor, hindering my ability to seek necessary information in a clear and organized manner.

[No.G2]

#### Behavioral Choices

Behavioral choice in older adults was influenced by two factors: information avoidance and information seeking. Specifically, those in the low-level declining and medium-level stable groups reported cognitive avoidance:

Given the uncertainty surrounding the accuracy of health information available on the internet, I continue to place my trust in my doctor’s guidance.

[No.D2]

Risk avoidance was reported by individuals in the medium-level stable group and the high-level declining group:

I felt immense panic due to the gravity with which this disease was portrayed on the internet.

[No.Z4]

Proactive information-seeking behavior was reported in the medium-stable group and the high-level declining group:

It’s really good to go online for health information, I’m able to look at diagrams and videos if I can’t read the text, and there are quite a few experts online.

[No.G2]

Those in the low-level declining group reported proxy information-seeking behavior:

I’m illiterate and have poor eyesight. My children handle my medical needs.

[No.D5]

Influencing factors for OHISB in the longitudinal survey were confirmed, explained, and supplemented in interviews with community-dwelling older adults

The integration of quantitative and qualitative results confirmed and supplemented the factors influencing OHISB at personal, environmental, and behavioral levels as presented in Multimedia Appendix 9. At the personal level, both methods identified DHL and TA as key factors. Qualitative findings further revealed subthemes—self-efficacy, risk perception, health anxiety, and self-perceived aging—grouped under personal cognition and emotional experience. While the quantitative study established pathways among DHL, TA, and OHISB, the qualitative study explained how personal factors shape search intentions and behaviors. At the environmental level, qualitative results on the influence of social support and the health care environment were validated by quantitative data, with qualitative insights further detailing support sources and environmental mechanisms. At the behavioral level, qualitative findings on diverse information behavior patterns explained the trajectory subgroups observed quantitatively. Together, both methods demonstrated that OHISB is dynamic, shaped by the interplay of personal and environmental factors.

## Discussion

### Principal Findings

This study used a longitudinal mixed methods approach to understand how older adults’ OHISB changes over time. We identified three distinct OHISB trajectories: low-level declining, medium-level stable, and high-level declining. Key factors influencing these paths included socioeconomic factors (eg, education, income), health status, internet use patterns, DHL, and TA. Importantly, TA mediated the longitudinal relationship between DHL and OHISB. Qualitative interviews highlighted four central themes—cognition, emotion, environment, and behavior—that help explain the differences across the three trajectory groups. These integrated findings provide a multi-faceted understanding of OHISB development in older adults.

LCGM identified three OHISB trajectories: medium-level stable group, characterized by sustained moderate engagement, aligning with German longitudinal findings on stable health information–seeking patterns among seniors [35]; low-level declining, reflecting digital avoidance due to ineffective retrieval, consistent with a phenomenon observed in Chinese older adults struggling with online health resources [36]; and high-level declining, where initial high engagement decreased, potentially linked to health anxiety’s bidirectional impact, suggesting that age-related health decline may amplify anxiety-driven disengagement [37]. The interviews explained the divergent trajectories through a pattern of information avoidance, particularly among older adults sensitive to content like disease prognosis, who actively avoided such negative information [38]. These findings align with the transtheoretical model of health behavior change [39], which posits behavior modification as a dynamic, stage-dependent process, as evidenced by similar trajectory heterogeneity in Chinese college students’ information-seeking patterns [12]. Critically, low-to-medium OHISB engagement prevailed, underscoring the urgency for community digital literacy programs and tailored policy interventions to ensure equitable access to online health resources.

The development of OHISB among community-dwelling older adults is shaped by multifaceted factors. Specifically, rural residence, low education or income, chronic diseases, and TA were identified as risk factors. Urban–rural resource disparities hinder digital access for rural older adults and worsen hollowing-out and empty-nest phenomena [40]. Integrated urban–rural development and increased rural investment are needed to bridge this digital divide. Higher education facilitates internet use and critical evaluation of health information, whereas those with less education rely more on traditional sources [41], indicating a role for community-based digital support. Socioeconomic advantages broaden internet access, and income positively predicts internet use, highlighting the urgency to address low-income seniors’ health needs [42]. Chronic diseases may trigger health anxiety–driven avoidance, requiring health care providers to offer psychological support and multisource health guidance. TA can hinder older individuals’ use of digital health services, leading to avoidance behaviors [43]. Simplifying digital health technologies and reducing the complexity of operations can lower anxiety levels and improve their willingness to use these services. Additionally, our interview results also found that suboptimal health care environments, such as complex offline care processes and poor service quality, impact OHISB [44]. When relegated to a passive role in medical interactions, older adults may proactively seek health information online to engage in medical decision-making [45], reflecting the dual influence of familial support and systemic health care barriers on health-seeking strategies. Furthermore, negative aging perceptions were linked to passive or avoidant OHISB, with self-perceived aging indirectly affecting OHISB via TA [46], underscoring health anxiety’s dual role and aging attitudes’ critical impact on digital health engagement.

Conversely, high frequency and duration of Internet usage, trust in online health information, prior online health-seeking experience, and higher DHL emerged as protective factors for OHISB. Greater internet exposure increases familiarity with online health resources, promoting active health information searches [47], whereas limited engagement exacerbates access barriers due to insufficient digital literacy and experience [48]. Community-based internet training and improved access can build foundational skills. Since trust determines information use [49], health care providers should promote online health services’ benefits and introduce trusted digital resources to shift perceptions. Meanwhile, positive experiences in finding useful health information online encourage older individuals to continue seeking [50], underscoring the need for user-friendly platforms and effective search training. Moreover, higher DHL enables efficient retrieval of quality information [51], supporting the value of targeted education. Qualitative findings indicate that high self-efficacy enables objective health or disease assessment, fostering proactive strategies and targeted searches for quality health information [52], consistent with prior evidence on self-efficacy’s positive impact on OHISB [53]. Additionally, high risk perception drives vigilance toward health threats and active use of online resources for disease prevention or management [54]. Notably, risk perception is moderated by risk attitudes: overload of negative health information may foster aversion to negative health information, thereby suppressing OHISB [55]. These findings highlight the interplay of cognitive resources (self-efficacy and risk perception) and contextual factors (information overload) in shaping older adults’ health-seeking behaviors. In addition, health anxiety showed a dual role: moderate levels promoted engagement [56] but excessive anxiety suppressed OHISB [57]. Lastly, family support enhances OHISB in community-dwelling older adults by boosting health awareness and perceived benefits [58].

This study demonstrates that DHL directly predicts OHISB and indirectly influences OHISB through TA. International studies corroborate the critical role of DHL in shaping OHISB. Older adults often experience anxiety due to concerns about technological complexity and privacy risks, though positive technological interactions can mitigate these effects and enhance device adoption. Notably, both perceived usefulness and interactive experiences depend on DHL. Grounded in the Technology Acceptance Model, Schiavo et al highlighted that attitudes toward new technologies are shaped by literacy and anxiety [59], with TA partially mediating this relationship. Cheng et al [60] provided a plausible explanation for the relationship among the three factors by introducing the stressor-strain-outcome model. In this model, DHL serves as a stressor, leading to technological stress, transitioning into TA, subsequently affecting individual performance, and resulting in negative usage behavior of online health services. These findings emphasize that enhancing OHISB requires not only improving DHL but also developing age-friendly platforms to reduce TA and optimize user experience.

### Limitations

This study has several limitations. First, reliance on self-reported data introduced potential response biases, which may compromise reliability of the results. Future studies should incorporate objective metrics to validate findings. Second, the focus on individual-level factors neglected environmental and technological determinants. A multidimensional framework integrating these elements could provide deeper insights. Third, the employment of convenience sampling could introduce selection bias and confine the generalizability of the results, highlighting the importance of employing more robust sampling approaches in upcoming research endeavors. Fourth, it should be noted that the cross-lagged panel model examined the relationships among DHL, TA, and OHISB without controlling for other potential confounding variables. Although this provides a clear test of the theoretical model, future studies with larger sample sizes could incorporate relevant covariates to further verify the robustness of these causal pathways. Fifth, as a mixed methods study, there were inherent challenges in effectively integrating quantitative and qualitative data, which may affect the depth of interpretation. Finally, the exclusive focus on Chinese older adults limited generalizability. Cross-cultural collaborations are needed to explore global applicability.

### Conclusions

This study identified three distinct trajectories of OHISB among older adults, revealing a concerning pattern of digital disengagement over time. The findings underscore that OHISB is not merely a technical challenge but is shaped by a complex interplay of cognitive, emotional, and socioeconomic factors, with TA playing a critical mediating role. These results call for a shift in policy and design thinking—from simply providing digital health resources to actively fostering digital inclusion. Interventions must be multifaceted, including tailored digital literacy programs, age-friendly designs that reduce anxiety, and policies that ensure equitable access. Ultimately, supporting older adults’ engagement with digital health is essential for achieving health equity in an increasingly aging and digital society.

## Supplementary material

10.2196/77549 Multimedia Appendix 1 The survey completion status of the older people in the community at three different time points.

10.2196/77549 Multimedia Appendix 2 Topic guide.

10.2196/77549 Multimedia Appendix 3 Comparison of baseline characteristics between participants who completed the follow-up and those who were lost to follow-up.

10.2196/77549 Multimedia Appendix 4 Model fitting indicators in the latent class growth modeling for online health information–seeking behaviors.

10.2196/77549 Multimedia Appendix 5 Parameters of the intercept and slope in the different developmental trajectories of online health information–seeking behaviors among the older adults in the community.

10.2196/77549 Multimedia Appendix 6 Results of differential testing in online health information–seeking behaviors among various trajectory subgroups.

10.2196/77549 Multimedia Appendix 7 Variable assignment.

10.2196/77549 Multimedia Appendix 8 General information about the interviewees.

10.2196/77549 Multimedia Appendix 9 Joint display of quantitative and qualitative findings.

## Abbreviations

BIC: Bayesian information criteria
DHL: digital health literacy
LCGM: latent class growth modeling
OHISB: online health information–seeking behavior
OR: odds ratio
TA: technology anxiety
