# Supplementary figures and images for: Trajectories and Influencing Factors of Online Health Information–Seeking Behaviors Among Community-Dwelling Older Adults: Longitudinal Mixed Methods Study
Source: J Med Internet Res. 2025 Nov 5;27:e77549. doi: 10.2196/77549 (PMC12588594; doi:10.2196/77549)

**
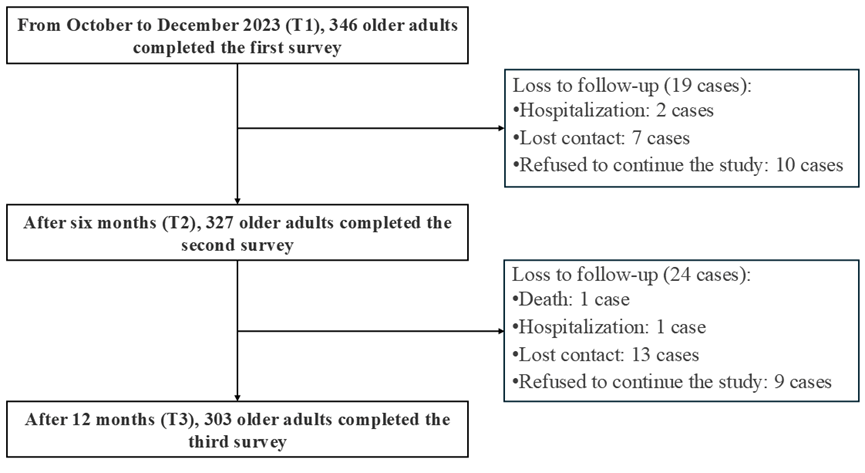
**

Supplement: Multimedia Appendix 1 [file jmir-v27-e77549-s001.docx]
