# Supplementary material for: Trajectories and Influencing Factors of Online Health Information–Seeking Behaviors Among Community-Dwelling Older Adults: Longitudinal Mixed Methods Study
Source: J Med Internet Res. 2025 Nov 5;27:e77549. doi: 10.2196/77549 (PMC12588594; doi:10.2196/77549)
Supplement: Multimedia Appendix 2 [file jmir-v27-e77549-s002.doc]

**Topic-guide for qualitative interviews**

1. What are your views on seeking health-related information?
2. What channels or sources do you use to access and search for health information?
3. How would you evaluate the health information available online?
4. How do you assess the practice of acquiring health information through the internet?
5. Do you choose to search for health information online? Why or why not?
6. What factors do you believe influence your own online health information-seeking behavior?
7. Have you ever browsed or searched for health information online? If so, please recall and describe the situation, your actions and feelings, as well as the impact it had on your life.
