# Supplementary material for: Trajectories and Influencing Factors of Online Health Information–Seeking Behaviors Among Community-Dwelling Older Adults: Longitudinal Mixed Methods Study
Source: J Med Internet Res. 2025 Nov 5;27:e77549. doi: 10.2196/77549 (PMC12588594; doi:10.2196/77549)
Supplement: Multimedia Appendix 3 [file jmir-v27-e77549-s003.docx]

| **Comparison of baseline characteristics between participants who completed the follow-up and those who were lost to follow-up** | | | | | |
| --- | --- | --- | --- | --- | --- |
| **Variable** | **Baseline participants**  **N=346**  **（%）/M±SD** | **Completed follow-up participants**  **N=303**  **（%）/M±SD** | **Lost follow-up participants**  **N=43**  **（%）/M±SD** | **χ^2^/t** | ***P*** |
| **Age** |  |  |  | 2.878^b^ | .237 |
| ≤65 years | 125(36.1) | 105(34.7) | 20(46.5) |  |  |
| 66～75 years | 169(48.8) | 153(50.5) | 16(37.2) |  |  |
| ＞75 years | 52(15.0) | 45(14.9) | 7(16.3) |  |  |
| **Gender** |  |  |  | 0.109^b^ | .742 |
| Male | 161(46.5) | 142(46.9) | 19(44.2) |  |  |
| Female | 185(53.5) | 161(53.1) | 24(55.8) |  |  |
| **Household registration** |  |  |  | 1.141^b^ | .285 |
| Urban | 203(58.7) | 181(59.7) | 22(51.2) |  |  |
| Rural | 143(41.3) | 122(40.3) | 21(48.8) |  |  |
| **Marital status** |  |  |  | 0.715^b^ | .499^a^ |
| Married | 324(93.6) | 285(94.1) | 39(90.7) |  |  |
| Never married / divorced / widowed | 22(6.4) | 18(5.9) | 4(9.3) |  |  |
| **Dwelling state** |  |  |  | 0.583^b^ | .847^a^ |
| Living with spouse | 299(86.4) | 263(86.8) | 36(83.7) |  |  |
| Living with children | 37(10.7) | 31(10.2) | 6(14.0) |  |  |
| Living alone | 10(2.9) | 9(3.0) | 1(2.3) |  |  |
| **Number of children** |  |  |  | 5.609^b^ | .061 |
| ≤1 | 70(20.2) | 65(21.5) | 5(11.6) |  |  |
| 2～3 | 180(52.0) | 160(52.8) | 20(46.5) |  |  |
| ≥4 | 96(27.7) | 78(25.7) | 18(41.9) |  |  |
| **Employment status** |  |  |  | 0.542^b^ | .559^a^ |
| Unemployed/job-seeking | 316(91.3) | 278(91.7) | 38(88.4) |  |  |
| Employed/ employed before retirement | 30(8.7) | 25(8.3) | 5(11.6) |  |  |
| **Education level** |  |  |  | 3.259^b^ | .353 |
| Primary school | 67(19.4) | 61(20.1) | 6(14.0) |  |  |
| Middle school | 147(42.5) | 130(42.9) | 17(39.5) |  |  |
| High school/vocational school/polytechnic school | 81(23.4) | 71(23.4) | 10(23.3) |  |  |
| College degree or above | 51(14.7) | 41(13.5) | 10(23.3) |  |  |
| **Income level** |  |  |  | 0.084^b^ | .959 |
| ＜2000 yuan/month | 66(19.1) | 58(19.1) | 8(18.6) |  |  |
| 2000～4000 yuan/month | 170(49.1) | 148(48.8) | 22(51.2) |  |  |
| ＞4000 yuan/month | 110(31.8) | 97(32.0) | 13(30.2) |  |  |
| **Medical insurance** |  |  |  | 0.658^b^ | .688^a^ |
| Employee resident medical insurance | 193(55.8) | 171(56.4) | 22(51.2) |  |  |
| Urban resident medical insurance | 142(41.0) | 122(40.3) | 20(46.5) |  |  |
| No | 11(3.2) | 10(3.3) | 1(2.3) |  |  |
| **Chronic disease status** |  |  |  | 0.598^b^ | .439 |
| Yes | 188(54.3) | 167(55.1) | 22(51.2) |  |  |
| No | 158(45.7) | 136(44.9) | 21(48.8) |  |  |
| **Self-assessment of health status** |  |  |  | 2.749^b^ | .253 |
| Very good/good | 96(27.7) | 86(28.4) | 10(23.3) |  |  |
| So so | 143(41.3) | 128(42.2) | 15(34.9) |  |  |
| Very bad/bad | 107(30.9) | 89(29.4) | 18(41.9) |  |  |
| **Degree of health concern** |  |  |  | 0.538^b^ | .764 |
| Concerned | 131(37.9) | 113(37.3) | 18(41.9) |  |  |
| Moderate | 111(32.1) | 97(32.0) | 14(32.6) |  |  |
| No concern | 104(30.1) | 93(30.7) | 11(25.6) |  |  |
| **Internet usage frequency** |  |  |  | 3.703^b^ | .295^a^ |
| Seldom | 40(11.6) | 38(12.5) | 2(4.7) |  |  |
| Occasionally | 126(36.4) | 107(35.3) | 19(44.2) |  |  |
| Sometimes | 137(39.6) | 122(40.3) | 15(34.9) |  |  |
| Often | 43(12.4) | 36(11.9) | 7(16.3) |  |  |
| **Internet usage duration** |  |  |  | 0.478^b^ | .489 |
| ＜5 years | 178(51.4) | 158(52.1) | 20(46.5) |  |  |
| ≥5 years | 168(48.6) | 145(47.9) | 23(53.5) |  |  |
| **Attitude towards online health information** |  |  |  | 4.994^b^ | .082 |
| Trust | 148(42.8) | 128(42.2) | 20(46.5) |  |  |
| Unsure | 103(29.8) | 86(28.4) | 17(39.5) |  |  |
| Distrust | 95(27.5) | 89(29.4) | 6(14.0) |  |  |
| **Willingness to seek online health information** |  |  |  | 0.179^b^ | .672 |
| Yes | 207(59.8) | 180(59.4) | 27(62.8) |  |  |
| No | 139(40.2) | 123(40.6) | 16(37.2) |  |  |
| **Experience in seeking online health information** |  |  |  | 0.240^b^ | .642 |
| Yes | 141(40.8) | 122(40.3) | 19(44.2) |  |  |
| No | 205(59.2) | 181(59.7) | 24(55.8) |  |  |
| **Digital Health Literacy** | 36.23±8.47 | 36.12±8.63 | 37.05±7.28 | -0.674^c^ | .501 |
| **Technology Anxiety** | 37.48±5.63 | 37.63±5.61 | 36.42±5.73 | 1.322^c^ | .187 |
| **Online Health Information-Seeking Behaviors** | 54.32±12.89 | 55.75±11.81 | 55.86±11.80 | -0.060^c^ | .953 |
| Note：a,adapt Fisher’s exact test; b, χ^2^; c,t. | | | | | |
