# Supplementary material for: Trajectories and Influencing Factors of Online Health Information–Seeking Behaviors Among Community-Dwelling Older Adults: Longitudinal Mixed Methods Study
Source: J Med Internet Res. 2025 Nov 5;27:e77549. doi: 10.2196/77549 (PMC12588594; doi:10.2196/77549)
Supplement: Multimedia Appendix 4 [file jmir-v27-e77549-s004.docx]

| **Model fitting indicators in LGCM for online health information seeking behaviors** | | | | | | | | |
| --- | --- | --- | --- | --- | --- | --- | --- | --- |
| Model | K | AIC | BIC | aBIC | Entropy | LMR（*P*） | BLRT（*P*） | Probability of class |
| C1 | 5 | 7650.831 | 7670.063 | 7654.202 |  |  |  |  |
| C2 | 8 | 7287.297 | 7318.069 | 7292.690 | 0.824 | .006 | *P*＜.001 | 0.263/0.737 |
| **C3** | **11** | **7027.390** | **7069.701** | **7034.805** | **0.861** | ***P*＜.001** | ***P*＜.001** | **0.266/0.540/0.194** |
| C4 | 14 | 6965.995 | 7019.845 | 6975.433 | 0.809 | .280 | *P*＜.001 | 0.124/0.280/0.431/0.165 |
| C5 | 17 | 6925.223 | 6990.613 | 6936.684 | 0.826 | .010 | *P*＜.001 | 0.442/0.142/0.208/0.116/0.092 |
| Note：K, Free estimation parameter; AIC, Akaike information criterion; BIC, Bayesian information criterion; aBIC, sample-size- adjusted BIC; LMR, Lo-Mendell-Rubin likelihood ratio test; BLRT, Bootstrap likelihood ratio test. | | | | | | | | |
