# Supplementary material for: Trajectories and Influencing Factors of Online Health Information–Seeking Behaviors Among Community-Dwelling Older Adults: Longitudinal Mixed Methods Study
Source: J Med Internet Res. 2025 Nov 5;27:e77549. doi: 10.2196/77549 (PMC12588594; doi:10.2196/77549)
Supplement: Multimedia Appendix 5 [file jmir-v27-e77549-s005.docx]

| **Parameters of the intercept and slope in the different developmental trajectories of online health information seeking behaviors among the elderly in the community** | | |
| --- | --- | --- |
| **Class** | **Intercept** | **Slope** |
| Low-Level Declining Group | 43.957*** | -2.159*** |
| Medium-Level Stable Group | 55.427*** | 0.309 |
| High-Level Declining Group | 73.743*** | -1.252* |
| Note：***P＜.001，*P＜.05 | | |
