# Supplementary material for: Trajectories and Influencing Factors of Online Health Information–Seeking Behaviors Among Community-Dwelling Older Adults: Longitudinal Mixed Methods Study
Source: J Med Internet Res. 2025 Nov 5;27:e77549. doi: 10.2196/77549 (PMC12588594; doi:10.2196/77549)
Supplement: Multimedia Appendix 6 [file jmir-v27-e77549-s006.docx]

| **Results of differential testing in online health information seeking behaviors among various trajectory subgroups** | | | | | |
| --- | --- | --- | --- | --- | --- |
| **Variable** | **Low-Level Declining Group N=92（%)/**  **M±SD** | **Medium-Level Stable Group N=187（%)/**  **M±SD** | **High-Level Declining Group N=67（%)/**  **M±SD** | **χ^2^/F** | ***P*** |
| **Age** |  |  |  | 14.099 | **.007** |
| ≤65 years | 24(26.1) | 79(42.2) | 22(32.8) |  |  |
| 66～75 years | 55(59.8) | 86(46.0) | 28(41.8) |  |  |
| ＞75 years | 13(14.1) | 22(11.8) | 17(25.4) |  |  |
| **Gender** |  |  |  | 0.145 | .930 |
| Male | 44(47.8) | 87(46.5) | 30(44.8) |  |  |
| Female | 48(52.2) | 100(53.5) | 37(55.2) |  |  |
| **Household registration** |  |  |  | 30.069 | **＜.001** |
| Urban | 37(40.2) | 110(58.8) | 56(83.6) |  |  |
| Rural | 55(59.8) | 77(41.2) | 11(16.4) |  |  |
| **Marital status** |  |  |  | 0.330 | .837^a^ |
| Married | 85(92.4) | 176(94.1) | 63(94.0) |  |  |
| Never married / divorced / widowed | 7(7.6) | 11(5.9) | 4(6.0) |  |  |
| **Dwelling state** |  |  |  | 4.802 | .308^a^ |
| Living with spouse | 76(82.6) | 162(86.6) | 61(91.0) |  |  |
| Living with children | 13(14.1) | 21(11.2) | 3(4.5) |  |  |
| Living alone | 3(3.3) | 4(2.1) | 3(4.5) |  |  |
| **Number of children** |  |  |  | 4.485 | .344 |
| ≤1 | 17(18.5) | 38(20.3) | 15(22.4) |  |  |
| 2～3 | 45(48.9) | 95(50.8) | 40(59.7) |  |  |
| ≥4 | 30(32.6) | 54(28.9) | 12(17.9) |  |  |
| **Employment status** |  |  |  | 13.893 | **＜.001** |
| Unemployed/job-seeking | 76(82.6) | 174(93.0) | 66(98.5) |  |  |
| Employed/ employed before retirement | 16(17.4) | 13(7.0) | 1(1.5) |  |  |
| **Education level** |  |  |  | 76.859 | **＜.001** |
| Primary school | 39(42.4) | 24(12.8) | 4(6.0) |  |  |
| Middle school | 43(46.7) | 87(46.5) | 17(25.4) |  |  |
| High school/vocational school/polytechnic school | 7(7.6) | 48(25.7) | 26(38.8) |  |  |
| College degree or above | 3(3.3) | 28(15.0) | 20(29.9) |  |  |
| **Income level** |  |  |  | 81.646 | **＜.001** |
| ＜2000 yuan/month | 38(41.3) | 27(14.4) | 1(1.5) |  |  |
| 2000～4000 yuan/month | 43(46.7) | 106(56.7) | 21(31.3) |  |  |
| ＞4000 yuan/month | 11(12.0) | 54(28.9) | 45(67.2) |  |  |
| **Medical insurance** |  |  |  | 8.367 | .076^a^ |
| Employee resident medical insurance | 42(45.7) | 107(57.2) | 44(65.7) |  |  |
| Urban resident medical insurance | 46(50.0) | 73(39.0) | 23(34.3) |  |  |
| No | 4(4.3) | 7(3.7) | 0(0.0) |  |  |
| **Chronic disease status** |  |  |  | 7.441 | **.024** |
| Yes | 39(42.4) | 108(57.8) | 41(61.2) |  |  |
| No | 53(57.6) | 79(42.2) | 26(38.8) |  |  |
| **Self-assessment of health status** |  |  |  | 7.903 | .095 |
| Very good/good | 31(33.7) | 47(25.1) | 18(26.9) |  |  |
| So so | 35(38.0) | 87(46.5) | 21(31.3) |  |  |
| Very bad/bad | 26(28.3) | 53(28.3) | 28(41.8) |  |  |
| **Degree of health concern** |  |  |  | 38.233 | **＜.001** |
| Concerned | 19(20.7) | 72(38.5) | 40(59.7) |  |  |
| Moderate | 26(28.3) | 68(36.4) | 17(25.4) |  |  |
| No concern | 47(51.1) | 47(25.1) | 10(14.9) |  |  |
| **Internet usage frequency** |  |  |  | 66.944 | **＜.001** |
| Seldom | 25(27.2) | 13(7.0) | 2(3.0) |  |  |
| Occasionally | 42(45.7) | 72(38.5) | 12(17.9) |  |  |
| Sometimes | 21(22.8) | 83(44.4) | 33(49.3) |  |  |
| Often | 4(4.3) | 19(10.2) | 20(29.9) |  |  |
| **Internet usage duration** |  |  |  | 77.530 | **＜.001** |
| ＜5 years | 78(84.8) | 90(48.1) | 10(14.9) |  |  |
| ≥5 years | 14(15.2) | 97(51.9) | 57(85.1) |  |  |
| **Attitude towards online health information** |  |  |  | 50.251 | **＜.001** |
| Trust | 14(15.3) | 88(47.1) | 46(68.7) |  |  |
| Unsure | 37(40.2) | 52(27.8) | 14(20.9) |  |  |
| Distrust | 41(44.6) | 47(25.1) | 7(10.4) |  |  |
| **Willingness to seek online health information** |  |  |  | 45.941 | **＜.001** |
| Yes | 30(32.6) | 121(64.7) | 56(83.6) |  |  |
| No | 62(67.4) | 66(35.3) | 11(16.4) |  |  |
| **Experience in seeking online health information** |  |  |  | 72.654 | **＜.001** |
| Yes | 14(15.2) | 72(38.5) | 55(82.1) |  |  |
| No | 78(84.8) | 115(61.5) | 12(17.0) |  |  |
| **Digital health literacy** | 28.12±6.615 | 37.17±6.297 | 44.76±5.903 | 139.279 | **＜.001** |
| **Technology anxiety** | 39.48±5.482 | 37.60±4.966 | 34.40±6.289 | 17.343 | **＜.001** |
