# Supplementary material for: Trajectories and Influencing Factors of Online Health Information–Seeking Behaviors Among Community-Dwelling Older Adults: Longitudinal Mixed Methods Study
Source: J Med Internet Res. 2025 Nov 5;27:e77549. doi: 10.2196/77549 (PMC12588594; doi:10.2196/77549)
Supplement: Multimedia Appendix 7 [file jmir-v27-e77549-s007.docx]

| **Variable Assignment** | | |
| --- | --- | --- |
| **Variable** |  | **Assignment** |
| **Dependent variable** | **Subgroup of Online Health Seeking Behaviors Trajectories** | Low-Level Declining Group=1;  Medium-Level Stable Group=2;  High-Level Declining Group=3; |
| **Independent variable** |  |  |
|  | **Age** | ≤65 years=1 (Z1=1, Z2=0);  66～75 years=2 (Z1=0, Z2=1);  ＞75 years=3 (Z1=0, Z2=0) |
|  | **Household registration** | Urban=1; Rural=2 |
|  | **Employment status** | Unemployed/job-seeking=0; Employed/ employed before retirement=1 |
|  | **Education level** | Primary school=1 (Z1=1, Z2=0, Z3=0);  Middle school=2 (Z1=0, Z2=1, Z3=0);  High school/vocational school/polytechnic school=3 (Z1=0, Z2=0, Z3=1);  College degree or above=4 (Z1=0, Z2=0, Z3=0) |
|  | **Income level** | ＜2000 yuan/month=1 (Z1=1, Z2=0);  2000～4000 yuan/month =2 (Z1=0, Z2=1);  ＞4000 yuan/month =3 (Z1=0, Z2=0) |
|  | **Chronic disease status** | No=0，Yes=1 |
|  | **Degree of health concern** | Concerned=1 (Z1=1, Z2=0);  Moderate=2 (Z1=0, Z2=1);  No concern=3 (Z1=0, Z2=0) |
|  | **Internet usage frequency** | Seldom=1 (Z1=1, Z2=0, Z3=0);  Occasionally=2(Z1=0, Z2=1, Z3=0);  Sometimes=3 (Z1=0, Z2=0, Z3=1);  Often=4 (Z1=0, Z2=0, Z3=0) |
|  | **Internet usage duration** | ＜5 years=1; ≥5 years=2 |
|  | **Attitude towards online health information** | Trust=1 (Z1=1, Z2=0);  Unsure=2 (Z1=0, Z2=1);  Distrust=3 (Z1=0, Z2=0) |
|  | **Willingness to seek online health information** | No=0; Yes=1 |
|  | **Experience in seeking online health information** | No=0; Yes=1 |
|  | **Digital health literacy** | Included as raw data |
|  | **Technology anxiety** | Included as raw data |
