# Supplementary material for: Trajectories and Influencing Factors of Online Health Information–Seeking Behaviors Among Community-Dwelling Older Adults: Longitudinal Mixed Methods Study
Source: J Med Internet Res. 2025 Nov 5;27:e77549. doi: 10.2196/77549 (PMC12588594; doi:10.2196/77549)
Supplement: Multimedia Appendix 8 [file jmir-v27-e77549-s008.docx]

| **General information about the interviewees** | | | | | | | | | | |
| --- | --- | --- | --- | --- | --- | --- | --- | --- | --- | --- |
| Number | Trajectory subgroups | Age | Gender | Household registration | Education level | Self-assessment of health status | Number of chronic diseases | Internet usage frequency | Attitude towards online health information | Experience in seeking online health information |
| D1 | C1 | 63 | female | Urban | Primary school | Bad | 2 | Occasionally | Unsure | No |
| D2 | C1 | 70 | male | Rural | Primary school | Good | 0 | Sometimes | Distrust | No |
| D3 | C1 | 68 | female | Urban | Middle school | So so | 1 | Occasionally | Unsure | Yes |
| D4 | C1 | 72 | male | Rural | Middle school | Good | 1 | Seldom | Distrust | No |
| D5 | C1 | 67 | female | Rural | Primary school | So so | 2 | Seldom | Distrust | No |
| Z1 | C2 | 64 | female | Urban | Primary school | Good | 0 | Occasionally | Unsure | No |
| Z2 | C2 | 60 | male | Rural | vocational school | Bad | 3 | Often | Trust | Yes |
| Z3 | C2 | 71 | female | Urban | Middle school | So so | 2 | Sometimes | Unsure | Yes |
| Z4 | C2 | 67 | male | Urban | Primary school | Good | 1 | Sometimes | Unsure | Yes |
| Z5 | C2 | 75 | male | Rural | Primary school | Bad | 1 | Occasionally | Distrust | No |
| Z6 | C2 | 65 | female | Urban | High school | So so | 0 | Occasionally | Trust | Yes |
| Z7 | C2 | 63 | female | Rural | Middle school | So so | 1 | Sometimes | Unsure | No |
| G1 | C3 | 62 | female | Urban | High school | good | 1 | Sometimes | Trust | Yes |
| G2 | C3 | 68 | male | Urban | polytechnic school | bad | 2 | Occasionally | Unsure | Yes |
| G3 | C3 | 65 | male | Urban | College | So so | 1 | Sometimes | Unsure | Yes |
| G4 | C3 | 73 | female | Rural | High school | So so | 1 | Often | Trust | Yes |

Note: C1, Low-Level Declining Group; C2, Medium-Level Stable Group; C3, High-Level Declining Group.
