# Supplementary material for: Trajectories and Influencing Factors of Online Health Information–Seeking Behaviors Among Community-Dwelling Older Adults: Longitudinal Mixed Methods Study
Source: J Med Internet Res. 2025 Nov 5;27:e77549. doi: 10.2196/77549 (PMC12588594; doi:10.2196/77549)
Supplement: Multimedia Appendix 9 [file jmir-v27-e77549-s009.docx]

| **Joint display of quantitative data and qualitative** | | | |
| --- | --- | --- | --- |
| Theoretical Domian | Quantitative Findings | Qualitative Findings | Summary of Integration |
| Personal Level | ①DHL and TA were influencing factors for OHISB among older adults in different trajectory subgroups；  ②Scores on online health literacy subscales of risk perception and health anxiety differed across trajectory subgroups；  ③DHL could directly predict OHISB among community-dwelling older adults and could also indirectly predict them through the mediation of TA. | ①Personal Cognition：Older adults in different trajectory subgroups exhibited distinct characteristics in health literacy, self-efficacy, and risk perception；  ②Emotional Experience：Older adults in different trajectory subgroups exhibited distinct characteristics in health anxiety, technological anxiety, and self-perception of aging. | Quantitative research explained the pathways between DHL, TA, and OHISB, providing data support for qualitative research. Qualitative research revealed themes not covered in the quantitative study, clarifying how individual factors influence seeking attitudes and subsequently behaviors, thus complementing the quantitative findings.  (Complementary) |
| Environmental Level | Scores on online health literacy subscales of social support and healthcare accessibility differed across trajectory subgroups. | ①Social support was an influencing factor for OHISB among older adults in different trajectory subgroups；  ②The offline healthcare environment influenced the OHIS behaviors of older adults in the "High-Level Declining" subgroup. | Qualitative research supplemented the sources of social support for community-dwelling older adults identified in the quantitative research and elaborated on how the healthcare environment influenced their OHIS behaviors.  (Convergent) |
| Behavioral Level | The OHISB of community-dwelling older adults were categorized into three groups: "Low-Level Declining," "Medium-Level Stable," and "High-Level Declining". | ①The information behaviors of community-dwelling older adults included both information seeking and information avoidance. Specifically, information seeking involved active seeking and proxy seeking, while information avoidance involved cognitive avoidance and defensive avoidance.；  ②The information behaviors of older adults in different trajectory subgroups overlapped. | Both qualitative and quantitative results indicated that the information behaviors of older adults are not fixed but are influenced by individual and environmental factors, presenting different behavioral patterns.  (Convergent) |

Note：DHL,Digital Health Literacy;TA, Technology Anxiety ;OHISB, Online Health Information Seeking Behaviors.
